# Effect of xenon and dexmedetomidine as adjuncts for general anesthesia on postoperative emergence delirium after elective cardiac catheterization in children: study protocol for a randomized, controlled, pilot trial

**DOI:** 10.1186/s13063-020-4231-5

**Published:** 2020-04-03

**Authors:** Sarah Devroe, Lisa Devriese, Frederik Debuck, Steffen Fieuws, Bjorn Cools, Marc Gewillig, Marc Van de Velde, Steffen Rex

**Affiliations:** 1 Department of Anesthesiology, University Hospitals Leuven, Herestraat 49, 3000 Leuven, Belgium; 2 Department of Pediatric and Congenital Cardiology, University Hospitals Leuven, Leuven, Belgium; 3 I-Biostat, KU Leuven – University of Leuven, Leuven, Belgium; 4 Department of Cardiovascular Sciences, Katholieke Universiteit (KU) Leuven, Leuven, Belgium

**Keywords:** Anesthetics, Inhalation, Xenon, Sevoflurane, Dexmedetomidine, Emergence delirium, Pediatric anesthesia

## Abstract

**Background:**

Emergence delirium, a manifestation of acute postoperative brain dysfunction, is frequently observed after pediatric anesthesia and has been associated with the use of sevoflurane. Both xenon and dexmedetomidine possess numerous desirable properties for the anesthesia of children with congenital heart disease, including hemodynamic stability, lack of neurotoxicity, and a reduced incidence of emergence delirium. Combining both drugs has never been studied as a balanced-anesthesia technique. This combination allows the provision of anesthesia without administering anesthetic drugs against which the Food and Drug Administration (FDA) issued a warning for the use in young children.

**Methods/Design:**

In this phase-II, mono-center, prospective, single-blinded, randomized, controlled pilot trial, we will include a total of 80 children aged 0–3 years suffering from congenital heart disease and undergoing general anesthesia for elective diagnostic and/or interventional cardiac catheterization. Patients are randomized into two study groups, receiving either a combination of xenon and dexmedetomidine or mono-anesthesia with sevoflurane for the maintenance of anesthesia.

The purpose of this study is to estimate the effect size for xenon-dexmedetomidine versus sevoflurane anesthesia with respect to the incidence of emergence delirium in children. We will also describe group differences for a variety of secondary outcome parameters including peri-interventional hemodynamics, emergence characteristics, incidence of postoperative vomiting, and the feasibility of a combined xenon-dexmedetomidine anesthesia in children.

**Discussion:**

Sevoflurane is the most frequently used anesthetic in young children, but has been indicated as an independent risk factor in the development of emergence delirium. Xenon and dexmedetomidine have both been associated with a reduction in the incidence of emergence delirium. Combining xenon and dexmedetomidine has never been described as a balanced-anesthesia technique in children. Our pilot study will therefore deliver important data required for future prospective clinical trials.

**Trial registration:**

EudraCT, 2018–002258-56. Registered on 20 August 2018. https://www.clinicaltrialsregister.eu.

## Background

Emergence delirium (ED) is a manifestation of acute postoperative brain dysfunction that occurs with a relatively high frequency after pediatric anesthesia. The incidence varies depending on the diagnostic criteria used and the combination of administered anesthetic drugs [1]. It can be associated with either short-term complications, such as an increased risk of injury in children, parental dissatisfaction, increased costs, and length of hospital stay, or potential long-term consequences, including persistent psychological and behavior problems [2]. While the exact pathophysiological mechanisms underlying ED remain unknown, the use of sevoflurane has been identified as one of the most important risk factors [3, 4] There is a continuous search for anesthetics that ameliorate or avoid the occurrence of emergence agitation [2].

In children with congenital heart disease (CHD), therapeutic decisions are often based on the hemodynamic parameters measured during cardiac catheterization. The ideal anesthetic technique for this procedure should maintain hemodynamics that reflect awake baseline conditions to provide the best therapeutic option. Unfortunately, most anesthetics affect myocardial function and the vascular tone, and the ideal anesthetic regimen for these procedures remains a subject of debate.

In adults, xenon anesthesia preserves baseline blood pressure (BP) better than volatile anesthetics or propofol [5]. Moreover, xenon has been repeatedly demonstrated to offer neuroprotection in various animal models and models of neuronal injury [6]. More specifically, xenon has been demonstrated to attenuate isoflurane-induced neurotoxicity in rats [7]. Whereas xenon can be used as a mono-anesthetic in adults, it can possibly only be used as an additive to other anesthetics in children. The minimum alveolar concentration (MAC) of xenon in children is unknown but, concurring with other anesthetics, is expected to be higher than in adults (MAC 63.1%) [8]. In a meta-analysis, the MAC of xenon for children at the age of 1 year was calculated to be 92% [9]. As a consequence, xenon can only be used in sub-anesthetic concentrations for children to guarantee an inspiratory oxygen concentration of at least 30%. While sub-anesthetic concentrations of xenon were demonstrated to be neuroprotective [10], they need to be supplemented by another anesthetic or sedative drug to achieve sufficient depth of anesthesia.

We previously demonstrated in children with CHD that the addition of 60% xenon to sevoflurane resulted in a 60% reduction of the mean expiratory sevoflurane concentration of sevoflurane to achieve a comparable depth of anesthesia [11, 12]. Moreover, we did find a decreased incidence of ED when xenon was added to sevoflurane anesthesia [11]. However, we could not demonstrate increased hemodynamic stability. Most probably, the hemodynamic advantages known from adults receiving mono-xenon anesthesia were masked by the necessary addition of sevoflurane to achieve an acceptable depth of anesthesia in children receiving xenon.

The ideal drug to add to xenon for balanced anesthesia should potentiate the anesthetic effects of xenon without jeopardizing hemodynamic stability.

Due to its sedative, anxiolytic, sympatholytic, and analgesic properties, dexmedetomidine has recently been introduced in different areas of pediatric anesthesia as an adjunct for balanced anesthesia, for premedication or as part of sedation techniques. Moreover, a recent systematic review and meta-analysis in pediatric patients undergoing congenital cardiac surgery found the administration of dexmedetomidine to result in more stable intraoperative hemodynamics, a lower incidence of ED, and an attenuated stress response [13–15]. A sparing effect of the MAC for sevoflurane has been demonstrated when an initial bolus of dexmedetomidine was followed by an infusion [16]. Moreover, the infusion of dexmedetomidine produced a dose-dependent decrease in the amount of sevoflurane required to produce 50% excellent tracheal intubation conditions in children [17]. Notably, like xenon, dexmedetomidine was also found to have neuroprotective properties in preclinical research. More specifically, it was observed that dexmedetomidine protects against anesthesia-induced neurotoxicity [18].

Altogether we can conclude that both xenon and dexmedetomidine possess numerous desirable properties with respect to the treatment of pediatric patients with CHD, including a benign hemodynamic profile [5, 14, 15], a reduced catecholamine release, anesthetic-sparing effects, a decreased incidence of ED, and neuroprotective effects [13]. This innovative combination of xenon and dexmedetomidine could be of particular interest for pediatric anesthesia, as combining these anesthetics has never been described as a balanced-anesthesia technique in children. Moreover, pediatric data on both drugs are scarce and, therefore, the FDA had no evidence to include these products in the “list of general anaesthetic and sedation drugs” for which a label change was requested indicating that “the repeated or lengthy use during surgeries or procedures in children younger than three years or in pregnant women during their third trimester may affect the development of children’s brains” [19].

## Methods/Design

### Aim of the study

The primary aim of this study is to describe the effect size for xenon-dexmedetomidine versus sevoflurane anesthesia with respect to the incidence of emergence delirium in children with CHD undergoing elective diagnostic/interventional cardiac catheterization. The secondary aim is to estimate group differences for a variety of secondary outcome parameters including peri-interventional hemodynamics, emergence characteristics, incidence of postoperative vomiting, and the feasibility of a combined xenon-dexmedetomidine anesthesia in children.

### Design of the study

The design of this study is reported in accordance with the SPIRIT 2013 Checklist: Recommended items to address in a clinical trial protocol (Additional file 1) [20].

This prospective, randomized, controlled, observer-blinded trial is performed at the University Hospitals of the KU Leuven by two types of investigators. Investigator I assesses the primary outcome (and the majority of the secondary outcome parameters) and is blinded to the group affiliation. Investigator II performs anesthesia and cannot be blinded to the treatment groups due to the mandatory monitoring of anesthetic concentrations. As a consequence, this study has to be performed observer-blinded.

The trial is conducted in compliance with the principles of the Declaration of Helsinki, the Principles of Good Clinical Practice, and in accordance with all applicable regulatory requirements. The protocol (version SD-DXP: 2 06-08-2018) and related documents were approved by the Ethics Committee of the University Hospitals Leuven (S61690 August 18th 2018) and the Federal Agency for Medicines and Health Products, Brussels, Belgium for Clinical Trial Authorization (FAGG/R&D/BEN/ben 1,111,021; 20 August 2018). The study is registered in the European Clinical Trails Database of the European Medicines Agency (EudraCT: 2018–002258-56) (see Additional file 2 for additional trial registration items).

To avoid selection bias, randomization and allocation concealment will be used. Patients are randomized to one of the study groups using a software-generated allocation sequence (RandList, V. 1.2). Group assignment is ensured by using sealed, opaque, sequentially numbered envelopes, which are opened only after arrival of the patient in the interventional room. As mentioned above, the attending anesthesiologist cannot be blinded for the treatment allocation. Consequently, a code break is not necessary.

### Inclusion and exclusion criteria

We screen all consecutive children scheduled for elective (diagnostic or therapeutic) heart catheterization under general anesthesia. We include children aged 1 month to 3 years.

Exclusion criteria comprise the lack of parental informed consent, a cyanotic congenital heart defect possibly requiring a FiO_2_ of > 50% during the procedure, high-risk and complex interventional procedures (as defined by the pediatric cardiologist), evidence of behavioral or cognitive impairment, and the presence of a contraindication for the use of one of the investigated drugs.

### Outcome parameters

#### Primary endpoint

- Incidence of ED as assessed by the Watcha-scale (4-point agitation scale) (Table 1) [21, 22]. A patient will be classified as having ED in case of a Watcha-scale ≥ 3. Moreover, every patient will also be tested using the “Pediatric Anesthesia Emergence Delirium Scale” (PAED scale) (Table 2). A patient will be classified as having ED in case of a PAED scale ≥ 10 [1, 15, 17]. In case of conflicting observations between the Watcha and the PAED scale, the results of the Watcha scale will be the crucial factor for the diagnosis of ED.

**Table 1:** WATCHA scale or 4-point agitation score [21]

WATCHA Scale	
Child is calm	1
Child is crying but can be consoled	2
Child is crying and cannot be consoled	3
Child is agitated and thrashing around	4

**Table 2:** PAED score [23]

PAED-scoring						
Point	Description of item	Not at all	Just a little	Quite a bit	Very much	Extremely
1	Child makes eye contact with caregiver	4	3	2	1	0
2	Child’s actions are purposeful	4	3	2	1	0
3	Child is aware of the surroundings	4	3	2	1	0
4	Child is restless	0	1	2	3	4
5	Child is inconsolable	0	1	2	3	4

#### Secondary endpoints

- Intraoperative hemodynamics:
  - ⚬ Heart rate (HR)
  - ⚬ Non-invasive BP (NIBP; systolic BP [SBP] and diastolic BP [DBP] and mean arterial pressure [MAP])
  - ⚬ Requirements of vasopressors, inotropes, chronotropes, and/or fluid boluses
- Incidence and duration of cerebral desaturation, defined as a decrease in regional cerebral tissue oxygenation (rScO_2_) of > 20% from baseline
- Feasibility parameters:
  - ⚬ Adequate depth of anesthesia as assessed with physiological signs (absence of movements, no rise in HR or BP of > 30% from baseline) and bispectral index (BIS) values
  - ⚬ Requirement of rescue medication to achieve an appropriate depth of anesthesia
  - ⚬ Intraoperative respiratory profile (pulse oximetry and capnography)
    - ▪ arterial oxygen saturation (SaO2)
    - ▪ end-tidal CO_2_
- Recovery parameters (measured from the stop of study treatment inhalation):
  - ⚬ Time to open eyes, time to extubation, time to Aldrete score ≥ 9 (readiness for discharge) [24]
  - ⚬ Recovery index: *RI=*$\frac{1+ Aldrete\ score\  at\ T5}{\left(2\bullet time\ to\ extubation\right)+ time\ to\ open\ eyes}$[25]
  - ⚬ Length of Post Anesthesia Care Unit (PACU) stay
  - ⚬ Length of hospital stay
- Other:
  - ⚬ Levels of serum protein S100β, IL-6, and IL-10 assessed at two time points (beginning and end of the procedure)
  - ⚬ Radiation dose
  - ⚬ Time of procedure

#### Safety endpoints

- ⚬ Incidence of postoperative vomiting (POV)
  - ▪ On the PACU
  - ▪ 12–24 h postoperatively
- ⚬ All other (serious) adverse events ((S)AE)

### Sample size

To date, the combination of dexmedetomidine and xenon has not yet been studied in humans. The present study is designed as a pilot trial with the aim of estimating the effect size for xenon-dexmedetomidine anesthesia versus sevoflurane anesthesia with respect to the incidence of ED. In a previous study by our group in a similar patient population, we observed a 40% incidence of ED in children receiving sevoflurane. We expect an accrual of 80 children in the planned study period of 2 years. With an inclusion of a total of 80 patients, we expect to have at least 35 children in each group when anticipating possible dropouts. It is assumed that the combination of xenon-dexmedetomidine is effectively reducing the incidence of emergence delirium to 20% (i.e. a 20% absolute risk reduction). This estimated effect size is based on own data comparing sevoflurane-xenon anesthesia with sevoflurane anesthesia [11] and on a recent meta-analysis reporting an odds ratio of 0.28 (95% confidence interval [CI] 0.19–0.40) for the effect of the intraoperative use of dexmedetomidine on the incidence of emergence delirium [26]. Based on an asymptotic 95% CI, the expected precision (half width of the CI) for the absolute risk reduction equals 21%, which is given by 1.96 times the standard deviation of the absolute risk reduction.

### Investigational plan and treatments

#### Preoperative treatment and monitoring

To objectively assess emergence parameters, the children receive no benzodiazepines for anxiolysis but the parents are encouraged to accompany their children until the induction of anesthesia. After arrival in the intervention room, the following monitoring is established and recorded every 5 min from the pre-anesthesia period to extubation: SaO_2_, NIBP (SBP-DBP-MAP), HR, F_i_O2, end-tidal O_2_ and CO_2_, temperature, rScO_2_, and BIS.

#### Induction of anesthesia

Anesthesia is induced with propofol 3 mg∙kg^− 1^, fentanyl 2 μg∙kg^− 1^, and rocuronium 0.3 mg∙kg^− 1^ as an intravenous bolus. Dexamethasone 0.15 mg∙kg^− 1^ and ondansetron 0.1 mg∙kg^− 1^ are given as standard POV prophylaxis. In the exceptional case that no intravenous line is available at induction, anesthesia is induced by mask-inhalation of sevoflurane in both study groups. In these cases, sevoflurane is immediately stopped once intravenous access has been achieved in group A (dex-xenon group).

#### Maintenance of anesthesia (anesthesia intervention)

Eligible children are randomly assigned to one of the two study groups:

- In group A (dex-xenon group), a loading dose of 1 μg∙kg^− 1^ of dexmedetomidine is administered at induction and anesthesia is maintained by a continuous infusion of dexmedetomidine (0.5–1.0 μg∙kg^− 1^∙h) and xenon 50%–65% in oxygen.
- In group B, anesthesia is maintained with sevoflurane alone (FiO_2_ = 0.25–0.4).

In group A, the infusion of dexmedetomidine and in group B the sevoflurane end-tidal concentrations are titrated to achieve physiological signs suggestive of an sufficient depth of anesthesia and BIS values in the range of 40–60.

#### Fluid management and postoperative analgesia

Fluid management is performed according to the “4/2/1-rule” (mL∙kg^− 1^∙h^− 1^) using a balanced crystalloid solution [27]. At the end of the procedure, all children receive paracetamol (15 mg∙kg^− 1^) for postoperative analgesia.

#### Standardized treatment of insufficient depth of anesthesia

In case of an insufficient depth of anesthesia, the dose of the maintenance anesthetic regimen is increased (xenon-dex group [group A]: dexmedetomidine infusion up to 1.2 μg∙kg^− 1^∙hr.^− 1^; sevo-group [group B]: increase of the age-corrected sevoflurane-concentration up to 1.5 MAC). If still no adequate plane of anesthesia can be achieved, a bolus of propofol (1–2 mg∙kg^− 1^) or fentanyl (1 μg∙kg^− 1^) is administered.

#### Standardized treatment of hemodynamic instability

Isolated blood pressure drops > 20% from baseline are treated with a bolus of phenylephrine (2–3 μg∙kg^− 1^) and/or a bolus of fluid (crystalloid 10 mL∙kg^− 1^); isolated HR declines with a bolus of atropine (10–20 μg∙kg^− 1^) and the combination of hypotension and bradycardia with a bolus of ephedrine (50–100 μg∙kg^− 1^).

### Study flow

#### Visit 0: Recruitment and baseline measurement visit

**Fig. 1:**
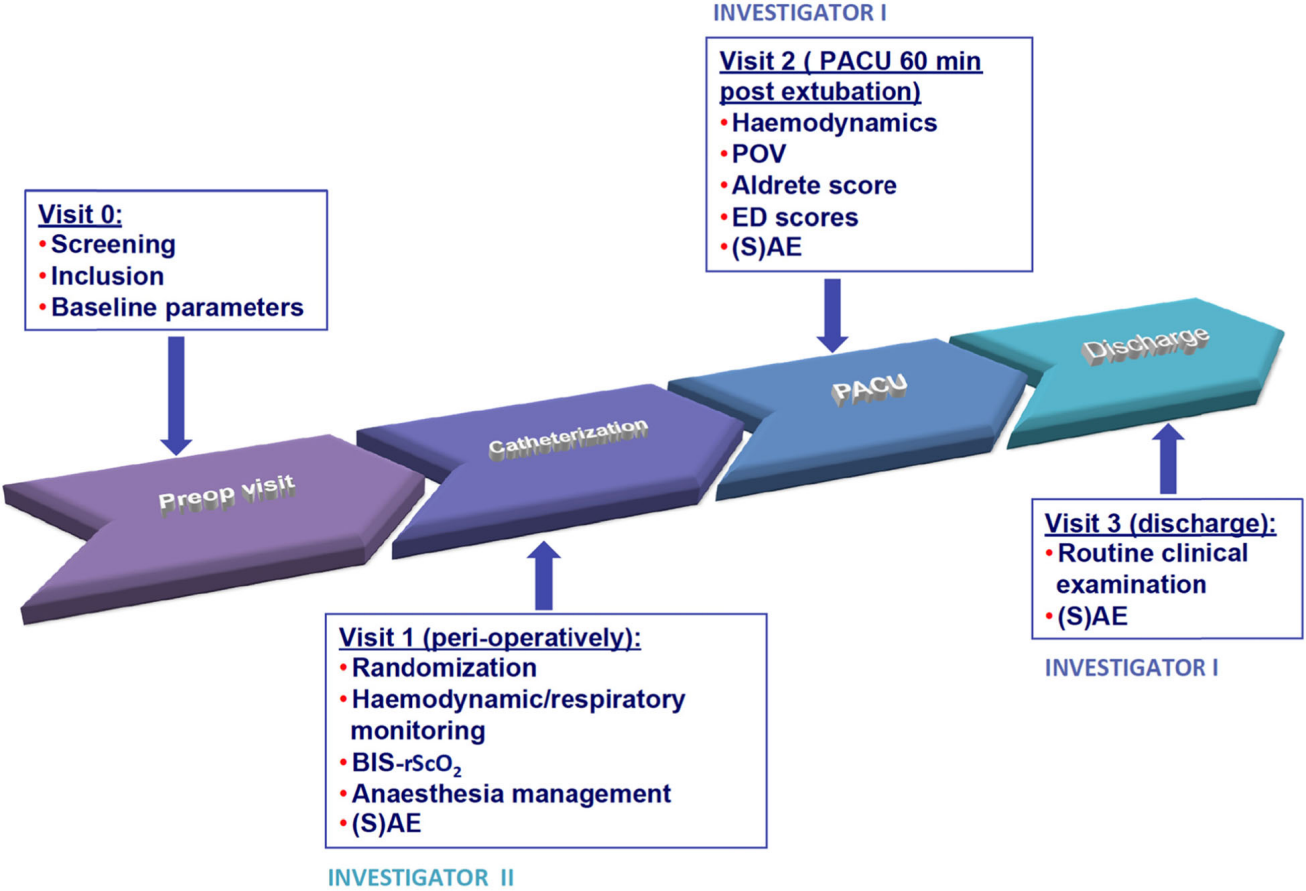
Schematic overview of the different steps of the study visits

**Fig. 2:**
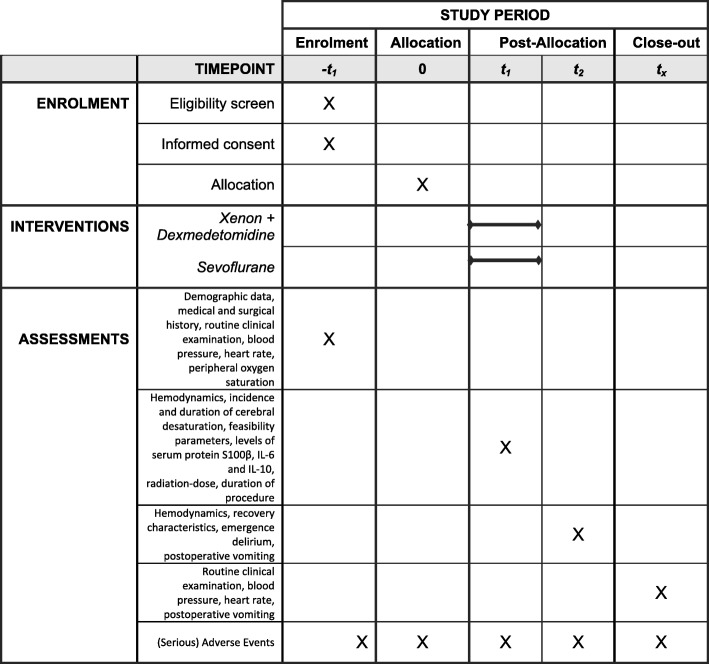
Schedule of enrolment, interventions, and assessments (SPIRIT)

After the obtainment of written parental informed consent (by one of the investigators), baseline data are evaluated and recorded (demographic data, medical and surgical history, routine clinical examination) (Figs. 1 and 2).

#### Visit 1: General anesthesia for cardiac catheterization by investigator II

General anesthesia will be induced and maintained as described above.

At the beginning and the end of the procedure, a blood gas sample and 2 mL of blood are obtained from the procedural sheet. After centrifugation, concentrations of S100β, IL-6, and IL-100 are determined using ELISA.

#### Visit 2: Post-anesthesia study visit PACU by investigator I

This visit is performed from the moment of extubation until 60 min after extubation and consists of the following assessments:

- Assessment of HR, BP, and peripheral oxygen saturation at 5, 10, 15, 30, 45, and 60 min after extubation.
- Assessment of POV at 5, 10, 15, 30, 45, and 60 min after extubation. An emetic episode is defined as a single or continuing occurrence of vomiting or retching. Distinct episodes are defined by an interval of respite of > 1 min. Rescue medication is offered once the patient has more than one emetic episode or on request of the patient or his parents ask.
- Aldrete score at 5, 10, 15, 30, 45, and 60 min after extubation.
- Assessment of emergence delirium by means of the “Four-point Agitation Scale” and the “PAED scale” at 5, 10, 15, 30, 45, and 60 min after extubation.
- Assessment of the incidences of (S)AEs.

#### Visit 3: Study end visit at the morning (12–24 h) after the procedure by investigator I

A routine clinical examination (including HR and BP) and the assessment of POV and (S)AE incidences are performed by investigator I on the day after the procedure.

### Data processing and statistical methodology

Intraoperative data are recorded manually every 5 min on specific case record forms (CRF). Regional cerebral tissue oxygenation (rScO_2_) is electronically recorded every second. Important pre- and postoperative data are manually documented during the pre-specified visits on CRFs. All data from the CRFs are anonymized and transferred into an electronic database (OpenClinica, LLC, Waltham, MA, USA). Direct access to source data and databases can be provided for trial-related monitoring, audits, EC review, and regulatory inspections.

All randomized patients will be included in the analyses. An investigator or a study nurse will review completed CRFs for completeness and correctness before digitalization and statistical analysis. Missing data will be identified, if possible drawn from source data, and filled into the CRFs. All results will be analyzed on an intention-to-treat basis. Statistical analysis will be performed using SPSS 23 software (SPSS Statistics for Windows, IBM, Armonk, NY, USA) or Prism® Software (Prism®, GraphPad Software, Inc., La Jolla, CA, USA). Since the aim of the study is exploratory, summary statistics (%, medians, means) and effect sizes with their 95% CI will be calculated. For the primary outcome and other binary variables, the absolute risk difference and its exact 95% CI will be reported; for normally distributed variables the difference in mean and a t-distribution-based 95% CI will reported; and for non-normal or ordinal variables the Hodges-Lehmann estimate with its 95% CI will be reported.

## Discussion

ED is a frequently observed condition following pediatric anesthesia as a consequence of which a child experiences emotional suffering, restlessness, and psychomotor disturbances. It can last up to 45 min, interferes with recovery, is associated with prolonged length of stay in the PACU and the hospital, and parental dissatisfaction. Additionally, ED can result in long-term maladaptive and behavioral disturbances (e.g. insomnia, fear, bed wetting, etc.) [2]. The diagnosis of ED remains notoriously difficult in young children. For several reasons, we opted in this study (as we did in several previous ones) for the Watcha scale as an instrument to assess the incidence of ED in this young population [11, 12, 28]. In fact, the frequently used PAED scale was derived and validated in a cohort of children with a mean age of 3.7 years [23]. In clinical practice, several items of the PAED (e.g. eye contact with the caregiver or awareness of the surrounding) appear very difficult to assess in neonates and infants. Interestingly, the latter items have recently been identified to be indicative for pain rather than for delirium [29]. Moreover, in a direct comparison of different scaling systems, the Watcha scale was found to be a more practical tool to assess ED in children after surgery and to have the highest overall sensitivity and specificity [22]. Note that the results of testing with the PAED scale will also be reported to allow comparison between these two instruments, an approach that is now increasingly be performed in the literature [4].

We specifically choose this patient population because: (1) the brains of these children are probably vulnerable to the adverse neuropsychiatric effects of sevoflurane; (2) younger age is a risk factor for ED; and (3) we observed a high incidence of ED in a population of children of the same age undergoing the same procedure [4, 11, 30].

Assessing the depth of anesthesia in children remains controversial. Moreover, the validity of the BIS monitor has never been formally tested in children undergoing combined anesthesia with xenon and sevoflurane. Available evidence suggests, however, that the BIS monitor is a suitable instrument to assess the depth of xenon anesthesia. In one study, during balanced xenon anesthesia, BIS values correlated well with the clinical assessment of hypnotic depth and were within the recommended range of adequate anesthesia [31]. Moreover, Stoppe et al. demonstrated that the BIS showed a similar response to xenon as to sevoflurane-anesthesia. In the latter study, the depth of anesthesia was additionally confirmed by the measurement of auditory evoked potentials [32]. Lastly, EEG changes observed during xenon anesthesia resembled closely those instigated by propofol [33]. Furthermore, although age-related changes in BIS values in children were observed, it has been suggested that the BIS is a reliable monitor of depth of anesthesia at least in children aged > 1 year, showing better performance with increasing age [34, 35]. Note that in our study, the adequacy of depth of anesthesia will primarily be assessed by physiological signs (absence of HR or BP increases and involuntarily movements) and the BIS monitor is only an additional tool to quantify depth of anesthesia.

We will assess the peri-interventional release of S100β as a secondary endpoint. The astroglial receptor protein S100β is involved in several cellular processes such as neuronal differentiation, axonal growth, and calcium homeostasis [36]. Increased levels of S100β in the peripheral blood have been suggested to indicate an increased permeability of the blood–brain barrier and found to be associated with neurotrauma, cerebral ischemia, cardiac arrest, and cardiac surgery [37]. In neonates suffering from CHD, levels of S100β were described to be inversely related to cerebral blood flow and to mortality [38]. In children undergoing general anesthesia for cardiac catheterization, we recently reported early neurocognitive deficits that were paralleled by an increase in S100β blood levels [12]. In adult patients undergoing cardiac surgery, high levels of S100β were observed to be correlated with adverse neuropsychological and psychiatric outcomes [39] and to predict the occurrence of postoperative delirium [36]. The exact mechanisms resulting in a perioperative/peri-interventional increase in S100β remain still unknown but might involve neuro-inflammatory pathways with astrocytes being stimulated by cytokines originating from systemic inflammation to release S100β. Therefore, we will also assess the peri-interventional release of the pro- and anti-inflammatory cytokines IL-6 and IL-10 in our population. We acknowledge that the findings will be exploratory, solely describe associations, and should not be used to assess causation.

The current standard of care for general anesthesia in childhood overall, and for cardiac catheterization more specifically, is sevoflurane. The use of sevoflurane has been linked to an increased incidence of ED [4]. Moreover, sevoflurane was listed by the FDA as potentially neurotoxic when used in children aged < 3 years (during the episode of rapid brain development). In contrast, dexmedetomidine and xenon have no proven neurotoxicity in preclinical research and even have the potential to reduce the incidence of ED [40]. The combination of both drugs has never been described as a balanced-anesthesia technique in children.

The exposure of young children to the combination of dexmedetomidine and xenon will provide the first human data of a pediatric anesthesia regimen without the use of any drugs that were indicated by the FDA to potentially impair neurodevelopmental outcome when repeatedly or lengthily used for general anesthesia and sedation during surgeries or procedures in children aged < 3 years or in pregnant women during their third trimester of pregnancy.

We acknowledge that our study is subject to several limitations. First, the attending anesthesiologist cannot be blinded for the interventional treatment. However, the primary outcome will be assessed by an investigator unaware of the treatment allocation (“observer blinded”). Second, it is not defensible to extrapolate the feasibility of xenon and dexmedetomidine as adjuncts for general anesthesia in young children undergoing cardiac catheterization to surgical settings with severe painful stimulation. Third, this randomized controlled trial is not designed to prove the potential superiority of xenon/dexmedetomidine versus sevoflurane anesthesia with regard to the incidence of ED. To perform a trial with this purpose, a precise idea of the expected ED incidences is necessary for appropriate sample size estimation. The current study will deliver data allowing to design an adequately powered randomized controlled trial addressing this research question. Note that the current study is also not sufficiently powered for all secondary endpoints, which should, therefore, be considered as purely explorative and hypothesis-generating.

In conclusion, this pilot trial will be performed to investigate the incidence of ED in an anesthesia regimen that does not involve sevoflurane. Moreover, we will study the feasibility and the safety of combined dexmedetomidine-xenon anesthesia in children.

### Benefits for the participating patients

There is no guarantee that the combination of dexmedetomidine and xenon, instead of standard treatment with mono-sevoflurane, will result in less ED or any other medical advantage to the participant.

### Safety issues

Standard hemodynamic monitoring in the setting of a fully equipped cardiac catheterization room enables immediate detection and treatment of AEs. Xenon inhalation or dexmedetomidine-infusion will be stopped immediately in case of a life-threatening deterioration of a study patient. All study participants will be closely monitored by the study team for the occurrence of (S)AEs. Moreover, the inclusion of each individual patient into the study is indicated in the electronic hospital information system and hence visible to all physicians and nurses involved in the care of this patient. This facilitates reporting of (S)AEs to the principal investigator. The principal investigator will report suspected unexpected serious adverse reactions (SUSARs) to the federal health authorities. Furthermore, study data will be regularly checked for safety by an independent clinical pediatric cardiologist who is not involved in this clinical trial. Lastly, the study is regularly audited by the clinical trials center of the sponsor (UZ Leuven).

### Data handling

All information and data relating to the study will be treated as confidential and such information will not be disclosed to any third parties or used for any purpose other than the performance of the study. The collection, processing, and disclosure of personal data such as patient health and medical information are in compliance with applicable European and national laws on personal data protection and the processing of personal data. Our data will be coded (continued link between the data and the individual who provided it). The participant’s name or other identifiers will be stored separately (in the investigators’ site file) and replaced with a unique code to create a new identity for the subject. Data will be entered into an electronic case record form (OpenClinica, Waltham, MA, USA). The study will be monitored by the clinical trials center of the UZ Leuven.

## Trial status

- Protocol version number and date: protocol SD-DXP 2 06-08-2018, approved by the ethics committee on 18 August 2018
- Date recruitment began: 18 December 2018
- Current status: 40 out of 80 patients have been recruited at the time of writing the manuscript
- Approximate date when recruitment will complete: 30 June 2020

## Supplementary information

**Additional file 1.** SPIRIT 2013 Checklist: Recommended items to address in a clinical trial protocol.

**Additional file 2.** World Health Organization trial registration dataset.

**Additional file 3.** Informed consent.

## Data Availability

Intraoperative data are recorded manually every 5 min on specific case record forms (CRF). Regional cerebral tissue oxygenation (rScO_2_) is electronically recorded every second. Important pre- and postoperative data are manually documented during the pre-specified visits on CRFs. All data from the CRFs are transferred into an electronic database (OpenClinica, LLC, Waltham, MA, USA). Direct access to source data and databases can be provided for trial-related monitoring, audits, EC review, and regulatory inspections. The datasets used and/or analyzed during the present study are available from the corresponding author on reasonable request.

## Abbreviations

BIS: Bispectral Index
BL: Baseline
CHD: Congenital heart disease
CRF: Case report form
DBP: Diastolic blood pressure
ED: Emergence delirium
FDA: United States Food and Drug Administration
F_i_O_2_: Fraction inspired oxygen
HR: Heart rate
IL: Interleukin
KU: Katholieke Universiteit
MAC: Minimal alveolar concentration
NIBP: Non-invasive blood pressure
OR: Odds ratio
PACU: Post Anesthesia Care Unit
PAED: Pediatric Anesthesia Emergence Delirium Scale
POV: Postoperative vomiting
RI: Recovery Index
rScO_2_: Regional cerebral tissue oxygenation
SBP: Systolic blood pressure
S_a_O_2_: Arterial oxygen saturation
(S)AE: (Serious) adverse event
SUSAR: Suspected unexpected serious adverse reaction
