# Supplementary material for: Effect of xenon and dexmedetomidine as adjuncts for general anesthesia on postoperative emergence delirium after elective cardiac catheterization in children: study protocol for a randomized, controlled, pilot trial
Source: Trials. 2020 Apr 3;21:310. doi: 10.1186/s13063-020-4231-5 (PMC7126401; doi:10.1186/s13063-020-4231-5)
Supplement: Supplementary file 2 — Additional file 2. World Health Organization trial registration dataset. [file 13063_2020_4231_MOESM2_ESM.docx]

**Additional file 2:** World Health Organization Trial Registration Data Set

| **Data category** | **Information** |
| --- | --- |
| Primary registry and trial identifying number | EudraCT: 2018-002258-56 |
| Date of registration in primary registry | August 20, 2018 |
| Secondary identifying numbers | NA |
| Source(s) of monetary or material support | Department of Anesthesiology, UZ Leuven |
| Primary sponsor | UZ Leuven, Herestraat 49, 3000 Leuven, Belgium |
| Secondary sponsor(s) | NA |
| Contact for public queries | Sarah Devroe, Department of Anesthesiology, UZ Leuven, Belgium |
| Contact for scientific queries | Sarah Devroe, Department of Anesthesiology, UZ Leuven, Belgium |
| Public title | The use of xenon and dexmedetomidine for the prevention of postoperative emergence delirium after anaesthesia for paediatric cardiac catheterization: A randomized, controlled, observer-blinded pilot trial. |
| Scientific title | The use of xenon and dexmedetomidine for the prevention of postoperative emergence delirium after anaesthesia for paediatric cardiac catheterization: A randomized, controlled, observer-blinded pilot trial. |
| Countries of recruitment | Belgium |
| Health condition(s) or problem(s) studied | Anesthesia for pediatric cardiac catheterization |
| Intervention(s) | General anesthesia with either the combination of xenon-dexmedetomidine or sevoflurane |
| Key inclusion and exclusion criteria | Inclusion Criteria:   - Age 0-3 years of age - Patient scheduled for elective (diagnostic or therapeutic) heart catheterization under general anesthesia   Exclusion Criteria:   - Lack of parental informed consent - Cyanotic congenital heart defects possibly requiring a FiO_2_ of > 50% during the procedure - High-risk and complex interventional procedures (as defined by the pediatric cardiologist) - Evidence of behavioral or cognitive impairment - Presence of contra-indication for the use of one of the investigated drugs:   - Dexmedetomidine:     - Hypersensitivity to the active substance or to any of the excipients.     - Advanced heart block (grade 2 or 3) unless paced.     - Uncontrolled hypotension.     - Acute cerebrovascular conditions.   - Xenon:     - Known history of hypersensitivity to the active substance.     - Susceptibility to malignant hyperthermia.     - Elevated intracranial pressure. |
| Study type | Allocation: Randomized Intervention Model: Parallel Assignment Masking: Observer-blinded Primary Purpose: Determination of effect size |
| Date of first enrolment | December 18, 2018 |
| Target sample size | 80 |
| Recruitment status | 40 out of 80 patients have been recruited at the time of writing the manuscript |
| Primary outcome(s) | Incidence of ED as assessed by the Watcha-scale (4-point-agitation scale). A patient will be classified as having ED in case of a Watcha-scale ≥ 3. |
| Key secondary outcomes | - Peri-interventional haemodynamics - Emergence characteristics - Incidence of postoperative vomiting - Feasibility of a combined xenon-dexmedetomidine anaesthesia in children |
