# Supplementary material for: Effect of xenon and dexmedetomidine as adjuncts for general anesthesia on postoperative emergence delirium after elective cardiac catheterization in children: study protocol for a randomized, controlled, pilot trial
Source: Trials. 2020 Apr 3;21:310. doi: 10.1186/s13063-020-4231-5 (PMC7126401; doi:10.1186/s13063-020-4231-5)
Supplement: Supplementary file 3 — Additional file 3. Informed consent. [file 13063_2020_4231_MOESM3_ESM.pdf]

Title of the study: The use of xenon and dexmedetomidine for the prevention of postoperative emergence delirium after anaesthesia for pediatric cardiac catherization: A randomized, controlled, observer-blinded pilot trial.

Research organisation: University Hospitals Leuven, Herestraat 49, 3000 Leuven

Medical Ethics Committee: *Identification of the Ethics Committee that issued the single opinion on the trial and the local Ethics Committee that took part in the approval process.*

Local investigators: *dr. Sarah Devroe and prof. dr. Steffen Rex, department of anesthesiology – University Hospitals Leuven, Herestraat 49, 3000 Leuven*

I Information vital to your decision to take part

Introduction

At this moment our hospital performs a clinical study concerning the use of xenon combined with dexdor for the prevention of postoperative emergence delirium after anaesthesia for pediatric cardiac catherization.

We would like to ask if your child may participate in this study.

Before you agree to take part in this study, we invite you to take note of its implications in terms of organisation, possible risks and benefits, to allow you to make a decision with full awareness of the implications. This is known as giving “informed consent”.

Please read these few pages of information carefully and ask any questions you want to the investigator or his/her representative. There are 3 parts to this document: the information essential to your decision, your written consent and supplementary information (appendices) detailing certain aspects of the basic information.

If you take part in this clinical study, you should be aware that:

- ➢ This clinical study is being conducted after having been reviewed by one or more ethics committees.
- ➢ Your participation is voluntary and must remain free from any coercion. It requires the signature of a document expressing your consent. Even after having signed this document, you can stop taking part by informing the investigator. Your decision not to take part or to stop taking part in the study will have no impact on the quality of your care or on your relationship with the investigator.
- ➢ The data collected on this occasion are confidential and your anonymity is guaranteed during publication of the results.
- ➢ Insurance has been taken out in case you should suffer any damage in connection with your participation in this clinical study.
- ➢ You will not incur any charges for the visits/consultations, examinations or treatments specific to this study.
- ➢ You may contact the investigator or a member of his/her team at any time should you need any additional information.

Further information about your “Rights as a participant in a clinical study” can be found in appendix III

Objectives and description of the study protocol

Antecedent clinical studies showed that the use of xenon is safe. Xenon anaesthesia can offer certain benefits in comparison to other anaesthetic products. For instance, xenon is quick-acting and leads to a faster recovery after the sedation. Also, xenon would have a less detrimental influence on the hemodynamic stability (fluctuations of blood pressure and pulse) during surgery. In newborns, the application of xenon resulted in a prominently stable cardiac rhythm and blood pressure. Furthermore it became apparent in various animal models that xenon protects the brain better than conventional narcotics against injury as a result of the intervention. Due to this less memory and concentration problems appear after the intervention. This protective effect could be beneficial for a brain in development, because this is probably more sensitive for the potential injury caused by the conventionalyl used narcotic devices. Currently, due to its protective properties, xenon is used for newly borns who suffered lack of oxygen before or during labour.

Because xenon is not powerful enough to be used as a single narcotic device for children, we should use it in combination with a second narcotic device. In this study we choose to combine xenon with dexdor. However dexdor is not yet approved for use in children, it is more often used for various paediatric indications. At our department, it is used as a short term sedation for radiological examinations (MRI-scan or isotope scan). Dexdor results in sedation, resolves anxiety and prevents agitation at waking up. Moreover, dexdor should have less effect on the hemodynamics (blood pressure and cardiac rhythm) and the respiration of the child compared to other narcotic devices.

Sevorane is the narcotic device that is usually used for the anaesthesia of children. An often occurring side-effect of sevorane is a highly agitated waking up. This is a terrifying side-effect for the parents and child itself. It can lead to a prolonged stay at the recovery room (at times children harm themselves) and behavioural disorders in the aftermath of the intervention (bed-wetting, fear of abandonment, sleeping difficulties)

In this study, we would like to evaluate if children wake up more calmly after anaesthesia with xenon and dexdor . Furthermore we want to assess if this sedation is at least equivalent to a sedation with Sevorane in the field of hemodynamic stability and depth of sedation. The potential effects on long term are not assessed during this study.

Course of the study

In this study 80 children in total will participate. The patients who participate, will be divided randomly in two groups (40 patients in each group) like heads or tails. Your child will receive anaesthesia with xenon and dexdor or with sevorane alone. Neither u, nor the physician at the consultation is informed of which group your child is classified in. In this way a more objective interpretation, a more scientific nature is guaranteed. The anaesthetist who will perform the sedation is aware of the group your child belongs to.

One day prior to the catheterization

Visit 0

Hereby you receive information regarding the study and read this informed consent form. The physician will answer all of your questions. In case you engage to participate in this study and sign this informed consent form, the physician makes sure you understand everything properly. After you have signed the informed consent form, the physician collects data from the medical file of your child. He/she will ask for information regarding the current medication use and possible interventions or treatments your child has received in the past.

Day of catheterization

Visit 1 (room where the intervention takes place)

At the day of the catheterization, your child will be divided in one of the two existing groups by chance: the group of xenon and dexdor anaesthesia or the group of sevorane anaesthesia. During the sedation, your child will be monitored non-stop. Every 5 minutes, a number of parameters are noted (such as heart beat, blood pressure). Likewise, the brain activity of your child will be continuously measured by EEG (to assure an adequate depth of the anaesthesia). Furthermore, the oxygen level of the brain will be observed by cerebral oxygen monitoring. When your child is asleep, the paediatric cardiologist will collect twice a small amount of blood (2ml) via catheter. This to determine the inflammatory response in the blood.

Visit 2 (1 hour post-op, recovery room)

A member of the research team will examine your son/daughter to verify if he/she experiences side effects of the anaesthesia or intervention (nausea, vomiting or distress). The way of waking up (calm or agitated) will be evaluated. One will make sure that the requirements for a return to the care-unit are all fulfilled.

Visit 3 (day 1 post-catheterization, usually the day of hospital discharge)

For the last time, the research team visits your child and will perform a routine medical examination. They also want to check for any belated side-effects of the anaesthesia or intervention (nausea, vomiting or distress).

Risks and discomforts

Modern anaesthesia is very safe, but side effects are always possible. Like any other medicine, xenon, dexdor as well as sevorane could have side effects. Notice that not each patient will experience these.

Possible side effects of xenon

- Very frequently occurring side-effects (more than 1 in 10 patients): increased blood pressure; nausea and vomiting
- Frequently occurring side-effects (less than 1 in 10 patients): increased body temperature and sweating; shivering, abnormal low cardiac rhythm; drops in blood pressure.

Possible side effects of dexdor

- Very frequently occurring side-effects (more than 1 in 10 patients): increased or decreased blood pressure; low heart rate
- Frequently occurring side-effects (less than 1 in 10 patients): fast heart rate; shift in breathing pattern; nausea and vomiting; agitation; fever.

Possible side effects of sevorane

- Very frequently occurring side-effects (more than 1 in 10 patients): agitation (unrest, frequently occurring in children); decelerated heart rate and fall of blood pressure; coughing (especially children); nausea and vomiting
- Frequently occurring side-effects (less than 1 in 10 patients): headache; fast pulse; high blood pressure; laryngospasm

We cannot guarantee that the administration of xenon in combination with dexdor instead of the standard anaesthesia which consist of sevorane alone, will offer your son or daughter any medical benefit. During the participation in this study, the physician will closely monitor the condition of your child. He/she will receive an adapted treatment at any moment. The information obtained thanks to this study may contribute to a better knowledge of the use of the combination xenon-dexdor in this intervention and may help future patients.

Which treatment does your child receive if it's not participating in this study?

Obviously, your decision not to participate in this study will have no influence on the following medical treatment in this hospital. Your child will receive the standard anaesthesia for this procedure. This standard anaesthesia is identical to the anaesthesia of group 2 in this study (anaesthesia via sevorane).

Samples of blood collected during the study

The blood samples will be coded and preserved by the investigator. The analysis of these samples will be performed after the completion of this study (expected time of accomplishment: December 31th 2019). These blood samples will be utilised exclusively for research objectives. There will be performed no other analyses than those carried out as part of this study without your consent and the permission of the Ethics Committee Research UZ/KU Leuven. You have every right to refuse supplementary examinations and are able to request elimination of all the samples. After the completion of the laboratory examinations and the registration of the results, the blood samples will be thrown away in accordance with generally valid measures. When you decide to withdraw from this study, u can claim the collected blood samples of your child or ask for their elimination. Please contact the investigator for this. The results based on the blood samples of your child which were obtained prior to your withdrawal of your consent to participate, remain property of the researcher.

Cessation of participation

Your participation is voluntary. You have every right to cease your participation based on any reason and without having to give any explanation.

Contact

If you need further information, but also if you have problems or concerns, you can contact the investigator (dr. Sarah Devroe) or a member of his/her research team (Huygens Christel) on the following telephone number (016/34 42 70).

If you have any questions relating to your rights as a participant in a clinical study, you can contact the patient rights ombudsman of your institution on this telephone number: 016/34 69 84. If necessary, he/she can put you in contact with the ethics committee. In case you have questions concerning the processing of your personal data , you can contact the UZ Leuven data protection officer (DPO) on this telephone number 016/34 69 84.

Title of the study: The use of xenon and dexmedetomidine for the prevention of postoperative emergence delirium after anaesthesia for pediatric cardiac catherization: A randomized, controlled, observer-blinded pilot trial.

II Informed consent

Participant

I declare that I have been informed of the nature of the study, its purpose, its duration, any risks and benefits and what is expected of my child. I have taken note of the information document and the appendices to this document.

I have had sufficient time to think about it and discuss it with a person of my choice, such as my GP or a member of my family.

I have had the opportunity to ask any questions that came to mind and have obtained a satisfactory response to my questions.

I understand that my participation in this study is voluntary and that I am free to end my participation in this study without this affecting my relationship with the therapeutic team in charge of my child's health.

I understand that data about my son or daughter will be collected throughout my participation in this study and that the investigator and the sponsor of the study will guarantee the confidentiality of these data in accordance with applicable European and Belgian legislation.

I agree the personal data of my son or daughter being processed as described in the section dealing with confidentiality guarantees (appendix IIII).

I agree/do not agree (delete as appropriate) to the study data collected for the purposes of this study being processed at a later date provided this processing is limited to the context of the present study for a better understanding of the disease and its treatment.

I agree to my GP or other specialists in charge of my health being informed of my participation in this clinical study.

I have received a copy of the information to the participant and the informed consent form.

____

Name patient (print letters)

__________________	_____________________	___/___/____
Name parent 1 (print letters)	Signature	Date
__________________	_____________________	___/___/____
Name parent 2 (print letters)	Signature	Date

Investigator

I, the undersigned, ___________________ investigator/clinical study assistant, confirm that I have verbally provided the necessary information about the study and have given the participant a copy of the information document.

I confirm that no pressure was applied to persuade the patient to agree to take part in the study and that I am willing to answer any additional questions if required.

I confirm that I operate in accordance with the ethical principles set out in the latest version of the "Helsinki Declaration", the "Good Clinical Practices" and the Belgian Law of 7 May 2004 related to experiments on humans.

_______________________	___________________	___/___/___
Name clinical study assistant who explained this study	Signature	Date
_______________________	___________________	___/___/___
Name investigator	Signature	Date

Title of the study: The use of xenon and dexmedetomidine for the prevention of postoperative emergence delirium after anaesthesia for pediatric cardiac catherization: A randomized, controlled, observer-blinded pilot trial.

III Supplementary information

Ethics Committee

This study has been reviewed by an independent Ethics Committee, namely the Ethics Committee which has issued a favourable opinion. It is the task of the Ethics Committees to protect people who take part in a clinical trial. They make sure that your rights as a patient and as a participant in a clinical study are respected, that based on current knowledge, the balance between risks and benefits remains favourable to the participants, that the study is scientifically relevant and ethical. The ethics committees give advice in accordance with the Belgian Law of 7 May 2004. You should not under any circumstances take the favourable opinion of the Ethics Committee as an incentive to take part in this study.

Voluntary participation

Before signing, do not hesitate to ask any questions you feel are appropriate. Take the time to discuss matters with a trusted person if you so wish.

Your participation in the study is voluntary and must remain free of any coercion: this means that you have the right not to take part in the study or to withdraw without giving a reason, even if you previously agreed to take part. Your decision will not affect your relationship with the investigator or the quality of your future therapeutic care.

If you agree to take part, you will sign the informed consent form. The investigator will also sign this form to confirm that he/she has provided you with the necessary information about the study. You will receive a copy of the form.

Costs associated with your participation

If you decide to take part in this study, this will not therefore involve any extra costs for you or your insurer. You may only be charged for the costs corresponding to the standard medical care in your child's clinical situation. You will not be compensated for participation in this study.

Guarantee of confidentiality

The data of your child will be processed in accordance with the European General Data Protection Regulation (GDPR). Your participation in the study means that you agree to the investigator collecting data about your child and using these data for research purposes and in connection with scientific and medical publications.

You are entitled to ask the investigator what data are being collected about your child and what is their use in connection with the study. This data concerns the current clinical situation of your child but also some of their background, the results of examinations carried out within the context of care of their health in accordance with the current standards and obviously the results of examinations required by the protocol. You have the right to inspect these data and correct them if they are incorrect[1].

The investigator has a duty of confidentiality vis-à-vis the data collected.

This means that he/she undertakes not only never to reveal the name of your child in the context of a publication or conference but also that he/she will encode (your child's identity will be replaced by an ID code in the study) the data of your child before sending them to the manager of the database of collected data

[1] These rights are guaranteed by the European Data Protection Regulation (GDPR), by the Belgian legislation on the protection of natural persons with regard to the processing of personal data and by the Law of 22 August 2002 on patient rights.

The investigator and his/her team will therefore be the only ones to be able to establish a link between the data transmitted throughout the study and the medical records of your child[2].

The personal data transmitted will not contain any combination of elements that might allow you to be identified[3].

For the study data manager designated by the sponsor, the data transmitted will not allow your child to be identified. The latter is responsible for collecting the data gathered by all investigators taking part in the study, processing them and protecting them in accordance with the requirements of the Belgian law on the protection of privacy.

To verify the quality of the study, it is possible that the medical records of your child will be examined by persons subject to professional secrecy and designated by the ethics committee, the sponsor of the study or an independent audit body. In any event, this examination of your medical records may only take place under the responsibility of the investigator and under the supervision of one of the collaborators designated by him/her.

The (encoded) study data will be able to be sent to Belgian or other regulatory authorities, to the relevant ethics committees, to other doctors and/or to organisations working in collaboration with the sponsor.

Your consent to take part in this study therefore also implies your consent to the use of the encoded medical data of your child for the purposes described in this information form and to their transmission to the aforementioned people and authorities.

If you withdraw your consent to take part in the study, to guarantee the validity of the research, the data encoded up to the point at which you withdraw will be retained. No new data will be collected.

If you have any questions relating to how the data of your child are being processed, you may contact the investigator. The data protection officer in your hospital can be contacted as well: DPO - UZ Leuven, Herestraat 49, 3000 Leuven, e-mail dpo@uzleuven.be.

Finally, if you have a complaint concerning the processing of your data, you can contact the Belgian supervisory authority who ensures that privacy is respected when personal data are processed.

The Belgian supervisory authority is called:
Data Protection Authority (DPA)
Drukpersstraat 35,
1000 Brussels
Tel. +32 2 274 48 00
e-mail: contact@apd-gba.be
Website: https://www.dataprotectionauthority.be

Insurance

Any participation in a clinical study involves a risk, however small it is. Even if there is no fault, the sponsor (UZ Leuven) accepts responsibility for damage caused to the participant (or in the event of death, his/her dependants) and directly or indirectly linked to his/her participation in the study. The sponsor has taken out insurance for this responsibility[4].

If the investigator believes that a link with the study is possible (the insurance does not cover the natural progression of the disease of your child or the known side effects of their normal treatment), he/she will inform the study sponsor, which will initiate the declaration procedure to the insurance company. The latter will appoint an expert - if it considers it necessary - to assess whether there is a link between your new health problems and the study.

In the event of disagreement either with the investigator or with the expert appointed by the insurance company and also whenever you feel it is appropriate, you or your dependants may bring proceedings

[2] For clinical trials, the law requires this link with your records to be retained for 20 years. In the case of a advanced therapy medicinal product using human biological material, this period will be a minimum of 30 years and a maximum of 50 years in accordance with the Belgian Law of 19 December 2008 on the use of human biological material and the applicable royal decrees.

[3] The database containing the results of the study will therefore not contain any combination of elements such as your initials, your gender and your full date of birth (dd/mm/yyyy).

[4] In accordance with Article 29 of the Belgian Law related to experiments on humans (7 May 2004)

against the insurer directly in Belgium (Amlin Europe NV, contract number 299.053.700, Vanbreda Risk & Benefits, Plantin Moretuslei 297, 2140 Antwerpen).

The law provides that the insurer may be summoned to appear either before the judge of the location where the event giving rise to the damage occurred, or before the judge of your domicile, or before the judge of the insurer's registered offices.
